# Challenges of concurrent HIV infection in the course and management of Crohn’s disease

**DOI:** 10.25122/jml-2024-0359

**Published:** 2025-03

**Authors:** Lamyaa Mattar, Husna Irfan Thalib, Meral Alnuwaimi, Hanin Alsaadi, Huda Ahmed Allouji, Jena Alyafei, Layan Alshowiman, Nuran Alsobyani, Fatma El Sayed Hassan

**Affiliations:** 1 Batterjee Medical College, Jeddah, Saudi Arabia; 2 Medical Physiology Department, Kasr Alainy Faculty of Medicine, Cairo University, Giza, Egypt; 3 Department of Physiology, Batterjee Medical College, Jeddah, Saudi Arabia

**Keywords:** HIV, Crohn’s disease, gut microbiota, probiotics, CD, Crohn’s Disease, IBD, Inflammatory Bowel Disease, GIT, Gastrointestinal Tract, UC, Ulcerative Colitis, NOD2, Nucleotide-Binding Oligomerization Domain–Containing Protein 2, CARD15, Caspase Recruitment Domain–Containing Protein 15, PRR, Pattern Recognition Receptor, NF-κB, Nuclear Factor Kappa B, IL, Interleukin, TNF-α, Tumor Necrosis Factor–α, DC, Dendritic Cells, APC, Antigen-Presenting Cells, CRC, Colorectal Cancer, SCFA, Short-Chain Fatty Acids, AIEC, Adherent-Invasive Escherichia Coli, ERS, Endoplasmic Reticulum Stress, CARD9, Caspase Recruitment Domain–Containing Protein 9, MAMP, Microbe-Associated Molecular Pattern, AAD, Antibiotic-Associated Diarrhea, PPAR-γ, Peroxisome Proliferator-Activated Receptor Gamma, TLR, Toll-Like Receptor, CD, Cluster Of Differentiation, NOS, Nitric Oxide Synthase, TGF-β, Transforming Growth Factor–β, SLE, Systemic Lupus Erythematosus, pDC, Plasmacytoid Dendritic Cells, HIV, Human Immunodeficiency Virus, ART, Antiretroviral Therapy, sCD14, Soluble CD14, FMT, Fecal Microbiota Transplantation, IFABP, Intestinal Fatty Acid–Binding Protein, FVT, Fecal Virome Transplantation, CAZymes, Carbohydrate-Active Enzymes, CCL4, C-C Motif Chemokine Ligand 4, ILCs, Innate Lymphoid Cells, CCR5, C-C Chemokine Receptor Type 5, NK, Natural Killer Cells, Th17, T Helper 17 Cells, CXCR4, C-X-C Chemokine Receptor Type 4, DC-SIGN, Dendritic Cell–Specific Intercellular Adhesion Molecule-3–Grabbing Non-Integrin, MALT, Mucosa-Associated Lymphoid Tissue, AIDS, Acquired Immunodeficiency Syndrome

## Abstract

Crohn’s disease (CD) is a chronic transmural bowel inflammation with a multifactorial etiology involving genetic predisposition and immune dysregulation in response to environmental triggers. In patients with human immunodeficiency virus (HIV), an already compromised immune system further complicates the progression and management of CD, creating unique therapeutic challenges. Probiotics have recently gained attention as a potential therapeutic option for CD, especially due to their role in modulating the gut microbiota. However, their effectiveness in patients with HIV, especially in enhancing and maintaining remissions, remains underexplored. This review aimed to examine how HIV infection influences the course of inflammatory bowel disease (IBD) and its impact on CD management strategies. A systematic literature search was conducted using Google Scholar, PubMed, Springer, and Web of Science to identify studies on patients with HIV and CD. HIV infection significantly alters the progression and management of CD due to its impact on the immune system. The immunosuppressed state of patients with HIV can complicate both the diagnosis and treatment of CD, often requiring adjustments in therapeutic approaches, necessitating a careful, tailored approach.

## INTRODUCTION

Inflammatory bowel diseases (IBDs) are chronic inflammatory conditions of the gastrointestinal tract (GIT) that affect millions of individuals worldwide. IBDs are primarily classified into Crohn's disease (CD) and ulcerative colitis (UC), both of which cause inflammation of the intestinal mucosa, manifesting as diarrhea, abdominal pain, rectal bleeding, and extra-intestinal manifestations like joint pain, skin lesions, eye inflammation, and liver disorders [1]. The global prevalence of IBDs continues to rise, posing significant economic and healthcare burdens, with 75% of studies on CD reporting a statistically significant increase in cases [2]. Sex-based differences in CD incidence have also been observed, with higher rates in men before age 15 and greater prevalence in women after 15, particularly beyond 40–44 years [3].

Managing CD in immunocompromised patients, such as patients with human immunodeficiency virus (HIV), is even more complex due to weakened immune systems [4]. Altered gut microbiota in these patients has sparked interest in using probiotics for treatment. When used alongside standard treatments, probiotics could provide added benefits in preventing CD relapses. This review examined the role of probiotics in managing CD in immunocompromised patients with HIV, focusing on gut microbiota and the potential of probiotics as a treatment option.

## MATERIAL AND METHODS

This literature review included studies published from January 2010 to January 2024. The databases used were Google Scholar, PubMed, Springer, and Web of Science using the keywords: 'Crohn’s disease', 'gut microbiota', 'probiotics', 'human immunodeficiency virus', and 'HIV'. Only English-language studies directly relevant to the theme of this review were included, while articles in other languages were excluded. A systematic approach to data extraction was employed to identify patterns, trends, and key findings, ensuring a comprehensive synthesis of the existing literature.

## PATHOGENESIS OF CD

**Figure 1:**
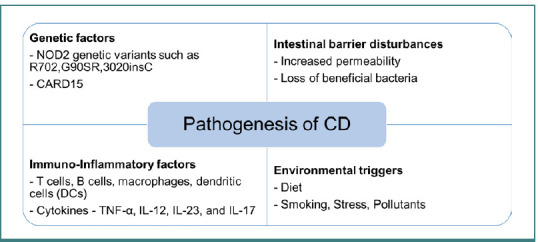
Pathogenesis of CD CD, Crohn's Disease; NOD2, Nucleotide-Binding Oligomerization Domain-Containing Protein 2; CARD15, Caspase Recruitment Domain-Containing Protein 15; DC, Dendritic Cells; TNF-α, Tumor Necrosis Factor-Alpha; IL, Interleukin

Disruption of the epithelial barrier and innate immunity defects lead to abnormal intestinal responses to harmless antigens in genetically vulnerable hosts. This encompasses three aspects: genetic factors, intestinal barrier disturbances, and immuno-inflammatory factors, as well as environmental triggers, as depicted in Figure 1 [4-6].

### Genetic factors

Two gene polymorphisms, *NOD2* and *CARD15*, participate in the innate immune response. *NOD2* detects bacterial wall components and controls immunity against intracellular bacteria in intestinal and immune cells. *NOD2* mutations reduce the ability to fight bacteria. *CARD15* regulates pro-inflammatory cytokines and protective molecules. *NOD2* is involved in maintaining intestinal homeostasis and is associated with an increased risk of developing CD. The most well-studied *NOD2* variants associated with CD are R702W, G908R, and 3020insC. Certain genetic variants of *NOD2* can impair immune responses to bacteria, causing reduced production of antimicrobial peptides, changes in cytokine secretion, and difficulty clearing intracellular bacteria. These defects can increase vulnerability to intestinal inflammation and the development of CD. The *NOD2* gene plays a crucial role in the pathophysiology of CD by regulating innate immune responses, maintaining intestinal balance, and impacting the relationship between host and gut microbiota. Genetic variants in *NOD2* can disrupt these functions, leading to aberrant immune activation and increased susceptibility to intestinal inflammation [7,8].

### Intestinal barrier disturbance

The intestinal epithelial barrier is a physical and immunological barrier between the gut lumen and the underlying tissues. There is evidence of impaired intestinal barrier function in CD, characterized by increased intestinal permeability, commonly referred to as a 'leaky gut' [9]. The intestinal barrier disruption allows toxins and antigens to enter the lining, leading to an immune system overreaction, tissue damage, and chronic inflammation. This imbalance weakens mucosal defenses and promotes bacterial invasion, and changes occur in the mucus layer and tight intercellular junctions [4,10,11].

### Immuno-inflammatory factors

CD involves dysregulated immune responses in which the body's immune system mistakenly attacks the GIT. Environmental triggers, such as dietary factors or microbial antigens, may stimulate an abnormal immune response in genetically susceptible individuals [12]. Key players include T cells, B cells, macrophages, dendritic cells (DCs), and cytokines such as tumor necrosis factor-alpha (TNF-α), interleukin-12 (IL-12), IL-23, and IL-17 [13]. In Peyer’s patches and lymphoid tissue, naïve T cells differentiate into effector T-helper (Th) cells after antigen-presenting cells (APCs) interact with bacterial antigens. This results in a cell-mediated immune response where Th1 cells induce APCs and macrophages to release a high concentration of IL-2, as well as an immune-mediated response where Th2 cells produce IL-4, IL-5, IL-6, IL-10, and IL-13 [4].

### Environmental triggers

Genetics predispose individuals to CD, but environmental factors like diet, smoking, stress, and exposure to certain microbes or pollutants can increase the risk and exacerbate symptoms. Smoking is a significant risk factor for CD, as it can exacerbate inflammation and alter the gut microbiota, leading to dysbiosis, alterations in immune function, increased intestinal permeability, and pro-inflammatory metabolites [14].

### Dysbiosis in CD

CD is characterized by inflammation in the GIT. While the precise causes of CD are not fully understood, emerging evidence suggests that dysbiosis plays a significant role in its development and progression. It includes three components: loss of beneficial bacteria, overgrowth of pathogenic bacteria, and loss of overall bacterial diversity [15].

### Loss of beneficial bacteria in CD

*Faecalibacterium prausnitzii (F. prausnitzii)* is one of the primary butyrate-producing bacteria in the intestine, playing a crucial role in gut physiology and overall host well-being. Butyrate serves as the main energy source for colonocytes and exhibits protective effects against colorectal cancer (CRC) and IBDs. Additionally, *F. prausnitzii* contributes to anti-inflammatory responses by promoting a tolerogenic cytokine profile characterized by increased secretion of IL-10 and minimal production of pro-inflammatory cytokines, such as IL-12 and interferon-gamma (IFN-γ) [16]. Similarly, *Bifidobacterium* species are helpful bacteria that ferment dietary fiber, produce short-chain fatty acids (SCFAs), and regulate immune reactions. They promote healthy guts and help maintain the intestinal barrier [17].

*Akkermansia muciniphila*, a mucin-degrading bacterium found in the human gut, plays a key role in thickening mucus and enhancing gut barrier function. Its metabolic activity produces short-chain fatty acids (SCFAs) that benefit the host and promote the growth of other beneficial microbiota. The precise causes of beneficial microorganism depletion in Crohn’s disease remain unclear. However, current evidence suggests a combination of genetic predisposition, environmental triggers, immune dysfunction, and alterations in the gut microbiome as contributing factors [18,19].

### Overgrowth of pathogenic bacteria and CD

Adherent-invasive *E. coli* (AIEC) strains are abundant in a subset of patients with ileal CD. These bacteria can invade, survive, and replicate in host cells, particularly inside macrophages, inducing high levels of TNF-α. This invasion triggers an immune response and causes chronic inflammation in the gut [20]. Similarly, *Fusobacterium nucleatum (F. nucleatum)*, a known pro-inflammatory bacterium, is frequently found in patients with CD. In CD progression, *F. nucleatum* triggers the endoplasmic reticulum stress (ERS) pathway, leading to the breakdown of the intestinal mucosal barrier. *F. nucleatum* causes damage to the mucosal barrier by directing its actions toward caspase activation and recruitment domain 3 (CARD3), which in turn triggers the ERS pathway [21-23].

### Loss of overall bacterial diversity and CD

A balanced gut microbiota is crucial for gut health, immune function, and preventing infections. Reduced microbial diversity leads to instability and increased susceptibility to pathogenic bacteria, which may contribute to CD pathogenesis [24]. Although a direct causal link between dysbiosis and CD has not been definitively established, accumulating evidence underscores the need to explore the microbial basis of CD at multiple levels, including microbial strain, genomic, epigenomic, and functional analyses, within specific clinical contexts. Moreover, mucosal immune dysfunction in CD is influenced by environmental factors such as diet, infections, medication use, stress, and smoking, which further shape gut microbiota composition and immune interactions [25].

### Factors modulating gut colonization in CD

The development of CD is influenced by a combination of genetic predisposition, gut microbiota imbalances, and an inadequate immune response; however, its exact etiology remains unclear [26]. Excessive immune response to luminal bacterial antigens causes tissue inflammation in CD. Intestinal mucus production is an innate defense mechanism against infections contributing to immune-mediated CD susceptibility. In mouse models, variations in the *MUC2* gene that reduce mucus production have been linked to CD. Interactions among these cells, integrins, adhesion molecules, and various chemokines lead to the release of inflammatory cytokines, which promote mucosal inflammation. In inflammatory bowel disease, especially CD, gut microbiota influences the inflammatory response. Specific microbial gene products can affect host gene expression, while microbe-associated molecular patterns (MAMPs) activate the immune response in chronic inflammation. The microbiome also provides data for metagenomics studies [27].

## PATHOGENESIS OF HIV

### HIV life cycle

HIV primarily targets cells that express CD4+ molecules, including macrophages, T-helper cells, and microglial cells. The virus binds to the CD4 receptor and chemokine co-receptors (CCR5 or CXCR4), facilitating fusion with the host cell membrane. Dendritic cells (DCs) express DC-SIGN, enhancing viral fusion with T cells during mucosal transmission. Once inside the cell, HIV uses reverse transcriptase and integrase to convert its RNA genome into complementary DNA (cDNA), which integrates into the host genome. This enables viral polyprotein synthesis, cleaved by furin protease into structural proteins for new virions. The virion then migrates to the cell membrane for the final step, which is budding [28,29].

### Host immune responses

Initially, CD4+ count increases as the innate immune system responds to the invading HIV. This early immune activation triggers the recruitment of CD8+ cytotoxic T cells, which target and destroy infected cells to control viral replication. CD4+ cell levels decline, particularly in the gut mucosal-associated lymphoid tissue (MALT), leading to compromised mucosal immune barrier integrity. This allows for microbial translocation, chronic immune activation, and systemic inflammation. Progressive CD4+ cell depletion weakens the immune system, making the host susceptible to opportunistic infections and cancers. If left untreated, this immune deterioration ultimately leads to the development of acquired immunodeficiency syndrome (AIDS), the most advanced stage of HIV infection [28].

### Dysbiosis in HIV-infected patients

**Figure 2:**
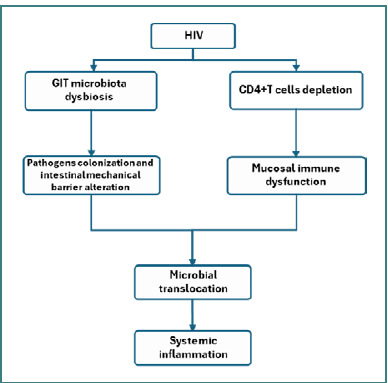
Gastrointestinal tract dysfunctions in patients with HIV

HIV infection is known to cause intestinal dysbiosis, though its specific effects on the gut microbiome require further study. The diverse microorganisms in the gut are essential for preventing bacterial invasion and maintaining microbial stability. Studies indicate that alpha diversity in the gut microbiome is significantly lower in patients with HIV [28,29]. With antiretroviral therapy (ART) initiation, CD4+ T cell counts typically recover, which is associated with increased alpha diversity. However, even after ART treatment, differences between HIV-infected individuals and healthy controls persist [29]. In patients with HIV, there is an increased presence of pathogenic opportunistic bacteria and a reduction in butyrate-producing bacteria and those with anti-inflammatory properties, compared to healthy individuals [30]. Research indicates that individuals with HIV have a higher abundance of harmful bacteria that promote inflammation and fewer bacteria that generate anti-inflammatory responses and SCFAs. For instance, the *Enterobacteriaceae* family is more prevalent in these patients, correlating with increased serum levels of soluble CD14 (sCD14), interleukin 1 beta (IL-1β), and interferon γ (IFNγ) [31]. These pathogenic bacteria may reduce CD4+ T cell count in the blood. HIV infection disrupts intestinal microbiota by increasing pro-inflammatory bacterial species and reducing those with anti-inflammatory functions, as depicted in Figure 2.

## CONSEQUENCES OF CD

In CD, vascular damage, focal arteritis, fibrin deposition, and arterial blockage in the muscularis propria lead to tissue infarction or neovascularization. Ongoing inflammation and immune activation may cause fibrosis, ulceration, and strictures in the intestinal wall [15]. These characteristics are absent in normal bowel and limited to affected areas. The findings suggest multifocal gastrointestinal infarction plays a role in mediating CD, with important implications for understanding the causes of the disease and enhancing treatment [16].

## THE EFFECT OF HIV INFECTION ON THE COURSE OF CD

A study of 195 patients with IBD, including 65 with HIV, examined the impact of HIV on IBD, noting effects on innate and adaptive immunity in the intestinal mucosa. HIV infection may shift immune cell balance by depleting Th17 and natural killer T (NK T) cells that express the CCR5 receptor while concurrently increasing regulatory T cells (Tregs). This shift in immune cell populations could potentially reduce IBD symptoms. Individuals with HIV often experience IBD remission and require less immunosuppressant therapy, possibly due to HIV’s influence on immune modulation. Both HIV-infected and uninfected patients showed similar IBD progression, suggesting HIV may reduce IBD symptom severity [32].

## CD TREATMENT

Treatment aims to manage inflammation and related complications such as fistula formation, obstructions, abscesses, and strictures to stabilize the condition. Additionally, treatment strategies aim to minimize the side effects of the regimens used to treat CD and the impact of the disease itself on patients’ lives [33,34].

### Dietary modifications

For children, the first line of management is dietary modifications that help reduce symptoms. Enteral nutrition may improve the condition of adult patients, based on small studies, and can serve as an alternative treatment method when medications are rejected or contraindicated [34].

### Medical treatment

**Figure 3:**
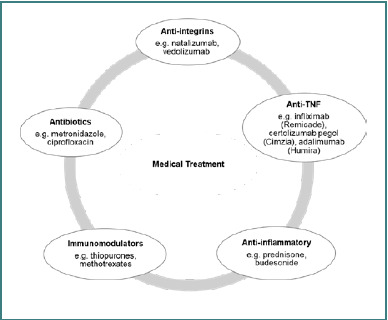
Medical treatment options for CD

Several factors determine the treatment choice for CD, such as age, symptoms, disease location and extent, inflammation status, comorbidities, and risk of more serious conditions, as shown in Table 1. When risk indicators suggest a poor prognosis or when the condition is severe, high-risk drugs are chosen. Over time, many advancements have improved treatment strategies [35,36]. Although definitive mucosal healing has yet to be consistently demonstrated, 5-aminosalicylates are commonly prescribed for mild to moderate symptoms [37]. For complications such as fistulas and abscesses, antibiotics are typically administered. Furthermore, corticosteroids, immunomodulators, and biologics play important roles in remission and stabilizing the condition, as illustrated in Figure 3.

**Table 1:** Factors affecting the choice of treatment in patients with CD [35,36]

Factor	Low risk	Moderate to high-risk
Age at initial diagnosis	> 30 years	< 30 years
Anatomic involvement	Limited	Extensive
Perianal or severe rectal disease	Absent	Present
Ulcers	Superficial	Deep
Stricture or penetrating behavior or Previous surgical resection	Absent	Present

### Corticosteroids

CD symptoms are commonly managed with corticosteroids. For severe cases, prednisone courses start at 40–60 mg, initially dosed at 5 mg and increased gradually to 20 mg, then adjusted by 2.5–5 mg weekly as needed. Prednisone is preferred for diffuse or left-colon disease, while budesonide (Entocort EC), with improved tolerability due to liver metabolism, is used for ileal and proximal colon involvement. Corticosteroids treat flare-ups but have adverse effects and are not effective for remission maintenance due to the risk of complications [37-39].

### Immunomodulators

Thiopurines and methotrexate are common immunomodulators in CD, though agents like azathioprine and 6-mercaptopurine are less effective as monotherapy. Immunomodulators act slowly but provide steroid-sparing effects. Combining azathioprine with anti-TNF agents like infliximab (Remicade) yields better results in moderate to high-risk patients, minimizing corticosteroid exposure, reducing adverse effects, and lowering anti-TNF immunogenicity [33-39].

### Biologics

The use of monoclonal antibodies for treating CD is approved and includes anti-TNF agents, anti-interleukin-12/23p40 antibodies, and anti-integrin therapies. These agents are administered initially to induce remission and must be continued for maintenance. Increased risk of certain cancers and infections, like tuberculosis reactivation, is associated with all monoclonal antibodies [34,39]. A recent meta-analysis showed that continuation of treatment with biologics is preferred over immunomodulatory therapy for enhanced effectiveness and tolerability [35]. In moderate to high-risk patients, or when corticosteroids and immunomodulators are less effective, anti-TNF agents like certolizumab pegol (Cimzia), adalimumab (Humira), and infliximab maintain remission. After 2 weeks of therapy initiation, the impact of the treatment must be detected. The effectiveness of anti-TNF agents is higher when induced within 2 years of disease onset [34].

### Probiotics in the treatment of CD

Probiotics are beneficial bacteria and yeasts that support health, many of which are already in the body. Probiotic supplements increase beneficial bacteria, enhance immunity, and fight harmful strains. Human gut microbiota is vital for intestinal development, homeostasis, and pathogen defense. Dysbiosis is linked to metabolic diseases (e.g., obesity, diabetes) and intestinal conditions, including antibiotic-associated diarrhea (AAD), CD, ulcerative colitis (UC), and colorectal cancer (CRC) [40]. A 2021 systematic review by Lopes *et al*. indicated that prolonged use of probiotics can help reduce IBD symptoms, leading to reduced intestinal inflammation and pro-inflammatory cytokines. However, further research is needed to determine optimal probiotic composition and duration of use due to significant variation in treatment approaches [41].

A 2018 study by Coqueiro *et al*. revealed that multiple probiotic strains effectively treat UC, a type of IBD, with *Lactobacillus* and *Bifidobacterium* being the most used. In some cases, probiotic supplementation may replace conventional therapy for UC, although probiotics for CD are controversial and not supported by results [42]. Similar findings were revealed in a 2014 study by Fujiya *et al*., which found probiotics beneficial in improving response and remission rates. Probiotics were as effective as mesalazine in maintaining UC remission but did not significantly affect inducing or maintaining CD remission [43]. A meta-analysis of nine clinical trials revealed that probiotics did not significantly impact CD overall but were effective in children with IBD. Certain combinations, such as *Saccharomyces boulardii, Lactobacillus*, and VSL#3 in CD, along with *Lactobacillus* and VSL#3 in children with IBD, showed promising results [44].

Probiotics modulate immune function and gastrointestinal flora to help maintain intestinal homeostasis. They stimulate antimicrobial peptide production and induce Paneth cells to secrete β-defensins. By competing with pathogens for nutrients and attachment sites on the epithelium, probiotics can also suppress pro-inflammatory cytokines, either by inhibiting the nuclear factor kappa B (NF-κB) pathway or by inducing peroxisome proliferator-activated receptor gamma (PPAR-γ). The combination of *Lactobacillus acidophilus, Bifidobacterium lactis, Lactobacillus plantarum*, and *Bifidobacterium breve*—formulated as Ultrabiotique—has also proven effective in a model of colitis, wherein treated mice had lower NF-κB, toll-like receptor (TLR)-4, and inducible nitric oxide synthase (iNOS). Probiotics, such as VSL#3, may enhance IL-10 while inhibiting IL-12 expression from favoring tolerogenic DC production via downregulating costimulatory molecules CD40 and CD80. Tolerogenic DCs promote the proliferation of immunosuppressive forkhead box P3+ Treg cells, which generate TGF-β and IL-10. In patients with systemic lupus erythematosus (SLE), *Lactobacillus delbrueckii* and *Lactobacillus rhamnosus* have been shown to generate tolerogenic DCs from monocytes. The interaction of intracellular TLR-9 in plasmacytoid DCs (pDCs) with unmethylated bacterial CpG DNA sequences produces type I interferon, a key factor in the anti-inflammatory effects of VSL#3 [45].

Vitamins are often delivered through diet. Vitamin D is mainly obtained from diet and sunlight exposure, yet over 80% of people in some countries are deficient. This deficiency has been linked to gut dysbiosis and inflammation, as vitamin D supplementation improves gut microbial diversity. In CD maintenance therapy, probiotics alone are ineffective in preventing clinical or endoscopic recurrence but may complement traditional treatments with mixed results. Some studies show post-surgery probiotic use can reduce inflammation, while others find no significant effect. Further research is needed to clarify the role of probiotics in CD treatment [46,47].

## TREATMENT OF PATIENTS WITH HIV AND GIT DISORDERS

### Narrow-spectrum antibiotics

Antibiotics are the primary treatment for bacterial infections. However, excessive use can lead to complications, including aggressive infections and multidrug-resistant bacteria. Additionally, antibiotics can disrupt intestinal bacterial homeostasis, contributing to dysbiosis [48]. For example, the narrow-spectrum antibiotic Ridinilazole has shown promising results in treating *Clostridioides difficile* infections with minimal impact on gut microbiota [49]. In contrast, excessive use of broad-spectrum antibiotics, such as tetracyclines, has caused significant damage to intestinal microbiota and reduced microbial diversity, even after treatment cessation [50]. For patients with HIV and low CD4+ T cell counts, trimethoprim-sulfamethoxazole is commonly prescribed as first-line treatment for Pneumocystis pneumonia; long-term use has a lesser impact on gut microbiota compared to other antibiotics [48,50]. Narrow-spectrum antibiotics are preferred to minimize disruption of gut microbiota, especially in patients with HIV and gastrointestinal disorders. For patients with IBD and *Clostridioides difficile* infection (CDI), narrow-spectrum antibiotics are recommended based on infection severity. For non-severe cases (white blood cell count ≤15,000 cells/mL and serum creatinine <1.5 mg/dL) and severe cases (white blood cell count >15,000 cells/mL and/or serum creatinine ≥1.5 mg/dL), the preferred treatment is oral vancomycin 125 mg four times daily for 10 days or fidaxomicin 200 mg twice daily for 10 days [51].

### Fecal microbiota transplantation (FMT) and fecal virome transplantation (FVT)

A recent study found that fecal microbiota transplantation (FMT) in patients with HIV increased gut microbiota alpha diversity and reduced levels of the intestinal injury marker, intestinal fatty acid-binding protein (IFABP). FMT improved intestinal dysbiosis and reduced intestinal damage in HIV infection [52]. Therefore, regulating intestinal microbiota should be part of HIV treatment. Fecal virome transplantation (FVT) is emerging as a promising alternative to FMT for treating pathogenic infections. FVT uses viral components from the gut virome, particularly bacteriophages, to manage intestinal dysbiosis and conditions like IBDs and alcoholic liver disease [53]. However, the use of bacteriophages to reduce inflammation in patients with HIV has not been reported. Future research should explore bacteriophage therapy in HIV management. The FMT exerts its therapeutic effect in IBD patients by restoring microbiome imbalances, utilizing specific compositions of donor stool. However, most clinical trials have focused on UC, and further studies are needed to clarify its role in CD remission. Additionally, FMT has been shown to increase SCFAs and secondary bile acids, which may contribute to its therapeutic effects [54].

### Personalized dietary regulation

For patients with CD and HIV, a balanced, low-fat, high-fiber diet is recommended. While high-fiber diets may not change gut bacteria variety, they boost enzymes that promote gut health [55]. Diets rich in fermented foods can increase gut bacteria diversity, reduce inflammation, and help regulate immune responses by influencing key immune cells like CD4+ T cells, CD8+ T cells, and B cells [56]. Additionally, ketogenic diets may increase beneficial bacteria like *Akkermansia* and reduce harmful bacteria such as Proteobacteria while lowering inflammation by reducing pro-inflammatory cytokines (IL-22, IL-17α, IL-18) and the chemokine CCL4 in colon tissues [57]. These dietary changes can support gut health, repair the gut lining, reduce harmful bacterial movement, and regulate inflammatory responses. Including these strategies in treatment may improve gut health and aid in managing both conditions.

### Probiotics in modulating immune response and managing dysbiosis in CD and HIV

Probiotics modulate the immune system and offer anti-inflammatory benefits. They treat HIV-induced dysbiosis and support IBD remission by adhering to the gut mucosa, competing for nutrients, inhibiting invasion, and stabilizing microbiota to protect against inflammation. Probiotics reduce mucosal and systemic inflammation, prevent microbial translocation, boost antimicrobial production, and strengthen the immunological barrier by enhancing intestinal IgA. In CD, probiotics show promise in restoring immune function and antioxidant defenses, mitigating HIV's impact on gut microbiota [58-60].

### Managing adverse effects of IBD treatment in patients with HIV

HIV-positive and negative patients had similar adverse effect rates from IBD therapies. Immunosuppressants caused side effects in 50% of HIV-positive patients, compared to 44.2% without HIV. For biologic therapies, 25.8% of HIV-positive patients experienced adverse events, similar to 30.4% in the uninfected group. These findings suggest that IBD treatments, including anti-TNF agents, are generally safe for HIV-positive patients. However, caution is needed as TNF-α affects HIV replication. Vedolizumab showed no adverse effects in HIV-positive individuals [32].

## CONCLUSION

CD is the result of a complex interaction between genetic predisposition, immune dysfunction, environmental components, and impaired intestinal barrier function. Probiotics are considered among the promising interventions for CD, but research is needed to establish the optimal strains, dosages, and treatment modalities. Management is complicated in patients with HIV since their immunity is already compromised. Although generally inexpensive treatments can improve intestinal health and symptoms, the efficacy of probiotics in this population is not established. Further studies are necessary to understand interactions between probiotics and immune dysfunction and to optimize formulations to improve clinical outcomes.

## PERSONAL THANKS

The authors thank Dr. Fatma EL Sayed Hassan for critically guiding and reviewing this article until publishing.
